# Supplementary figures and images for: Moesin controls cell–cell fusion and osteoclast function
Source: J Cell Biol. 2025 Oct 27;224(11):e202409169. doi: 10.1083/jcb.202409169 (PMC12558046; doi:10.1083/jcb.202409169)

WB\_Figure 2A-B

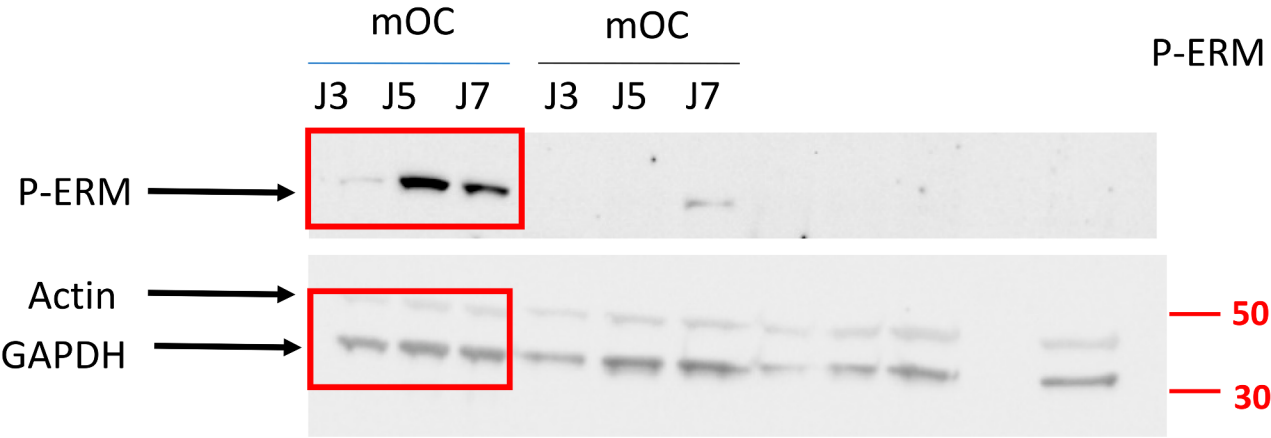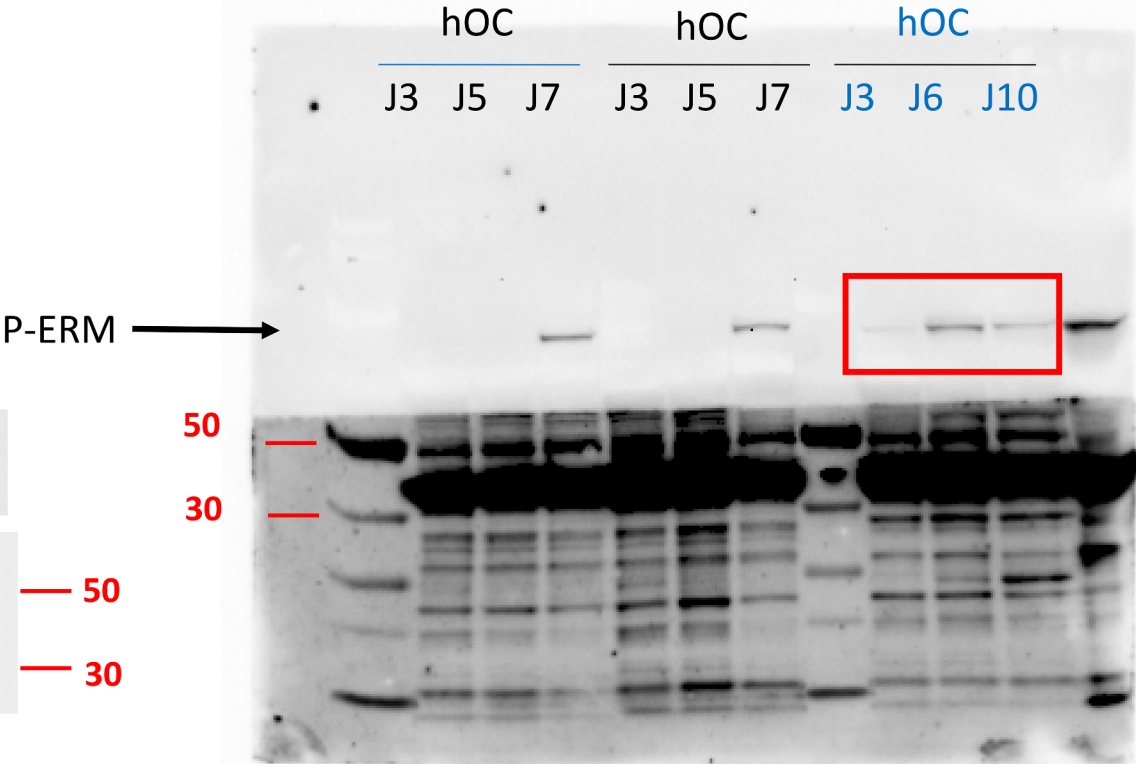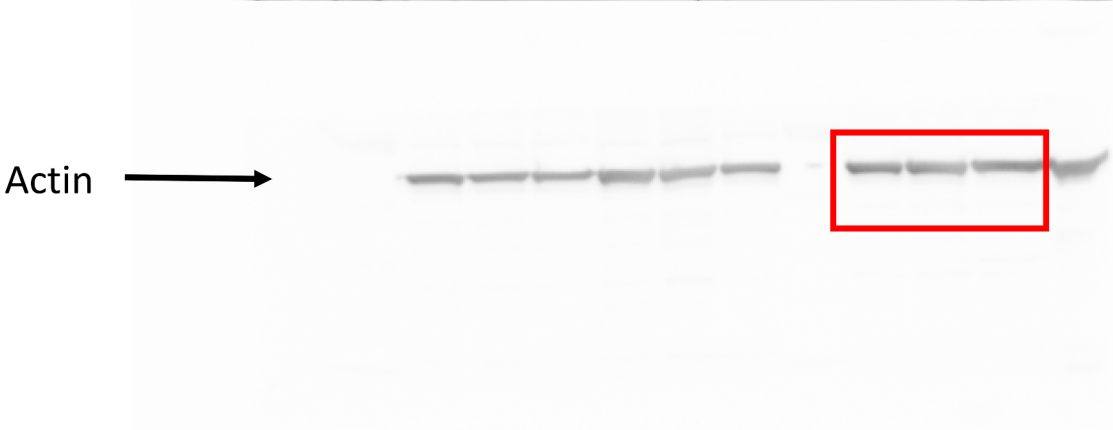

WB\_Figure 2F

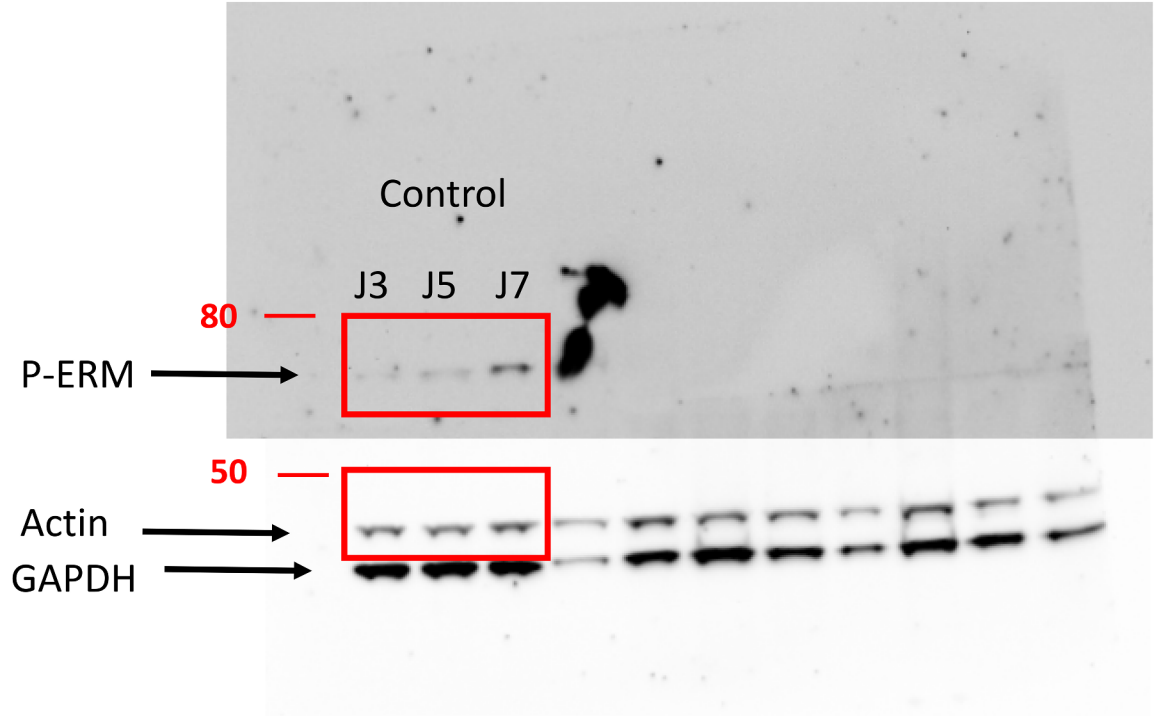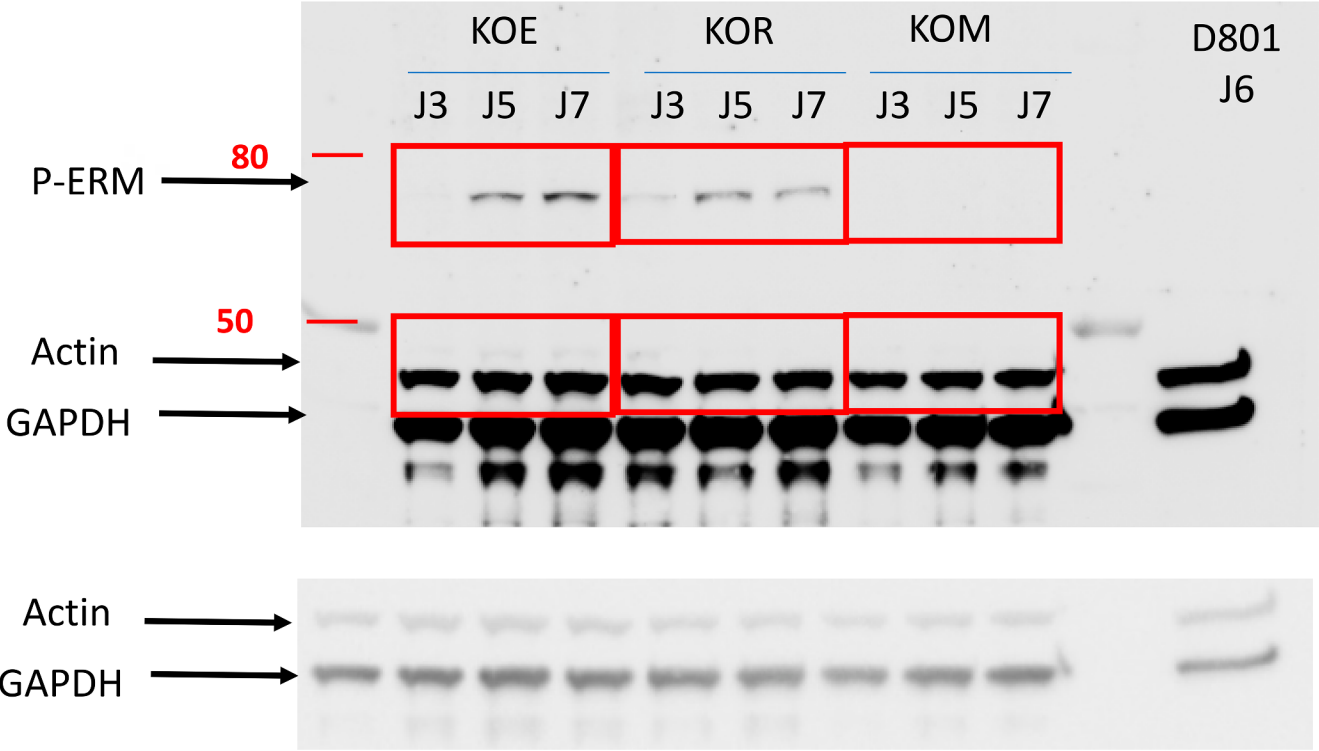

Supplement: SourceData F2 — is the source file for Fig. 2. [file jcb_202409169_sourcedataf2.pdf]

WB\_Figure 5C

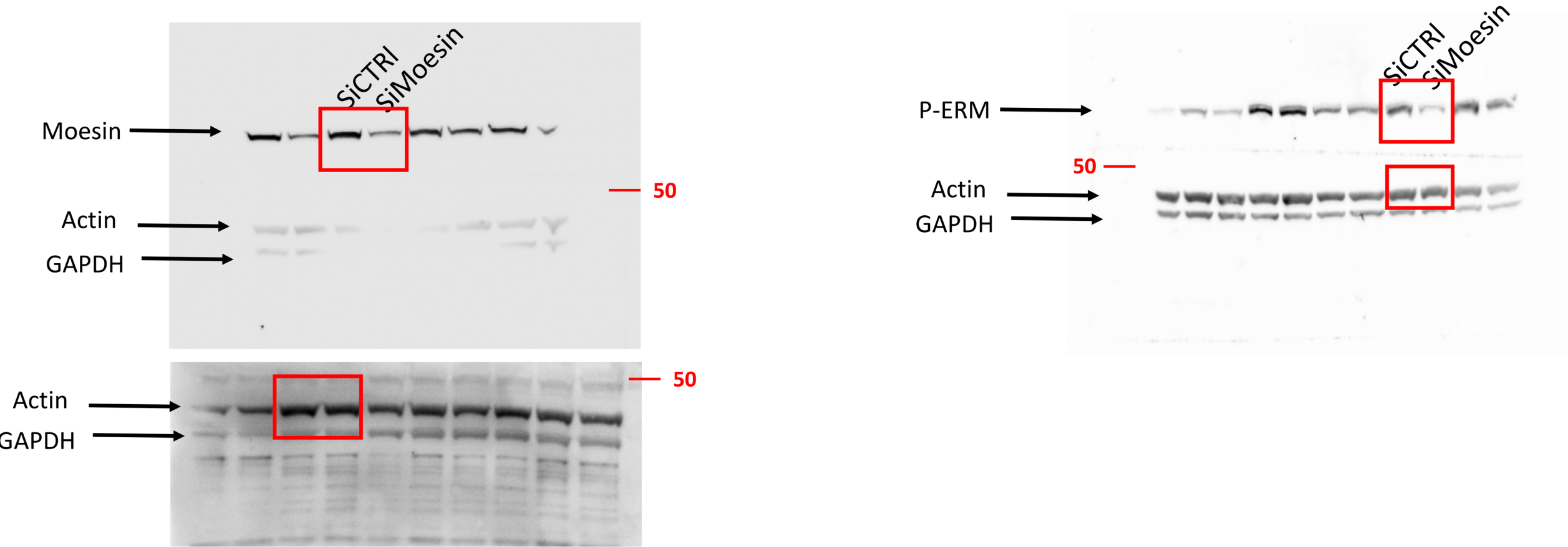

Supplement: SourceData F5 — is the source file for Fig. 5. [file jcb_202409169_sourcedataf5.pdf]

WB\_Figure 6A

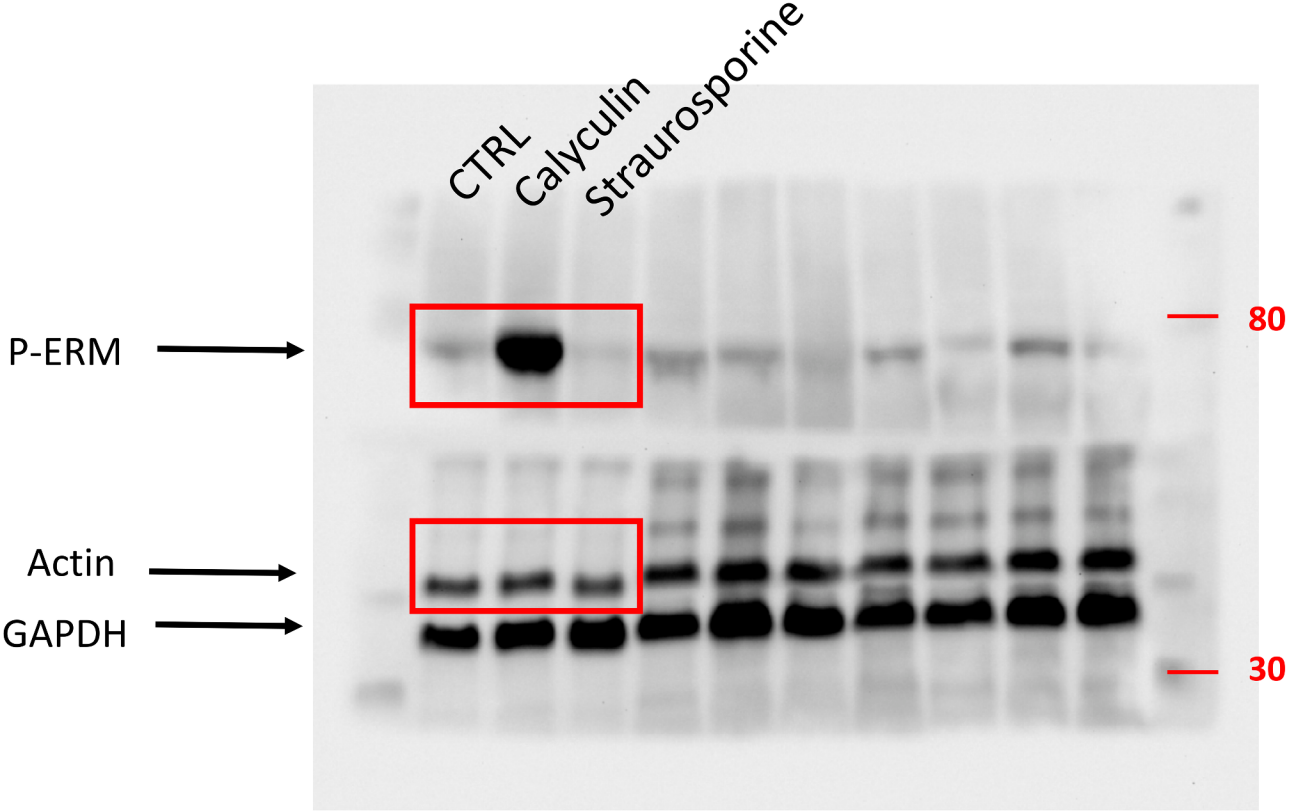

WB\_Figure 6B

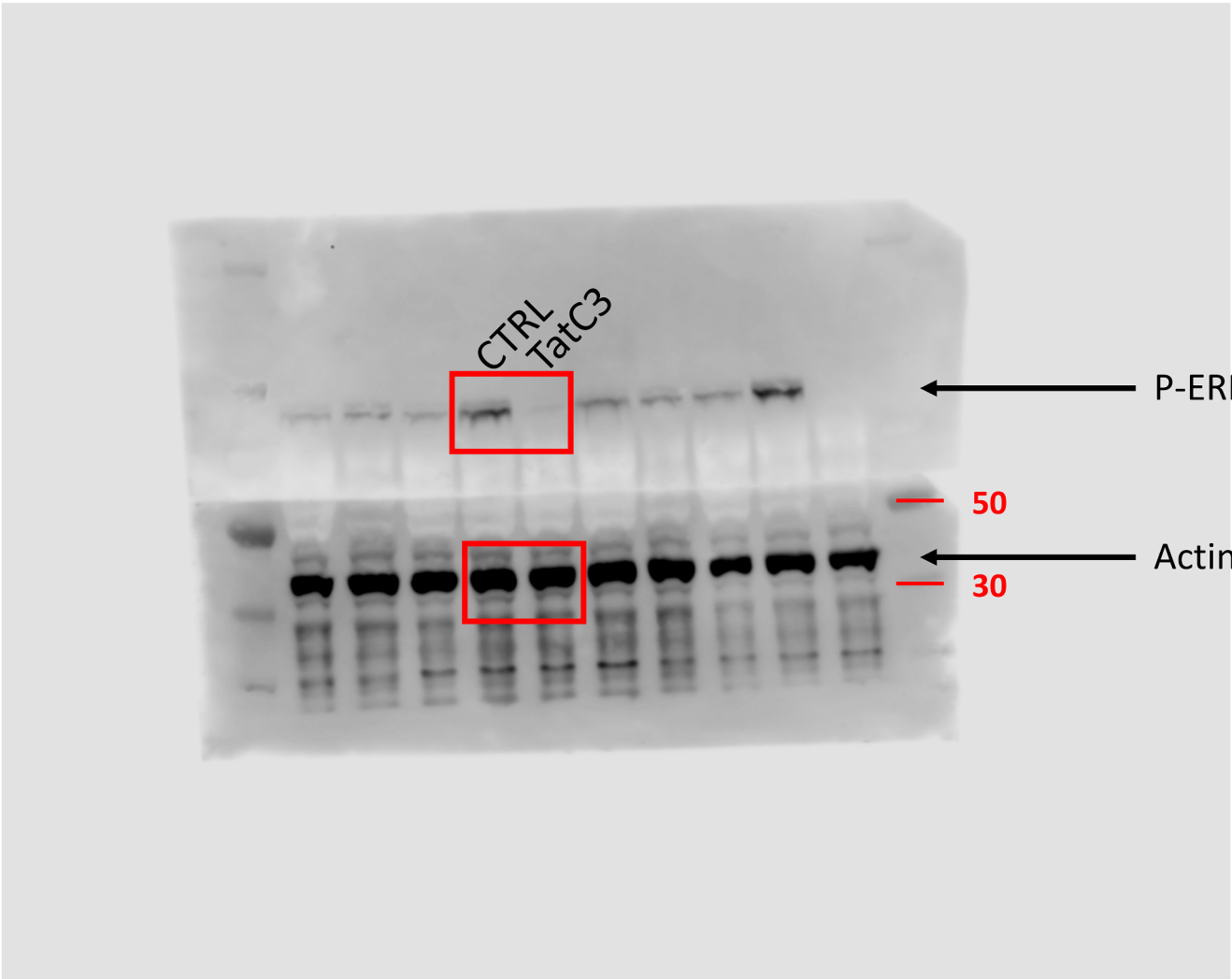

WB\_Figure 6C

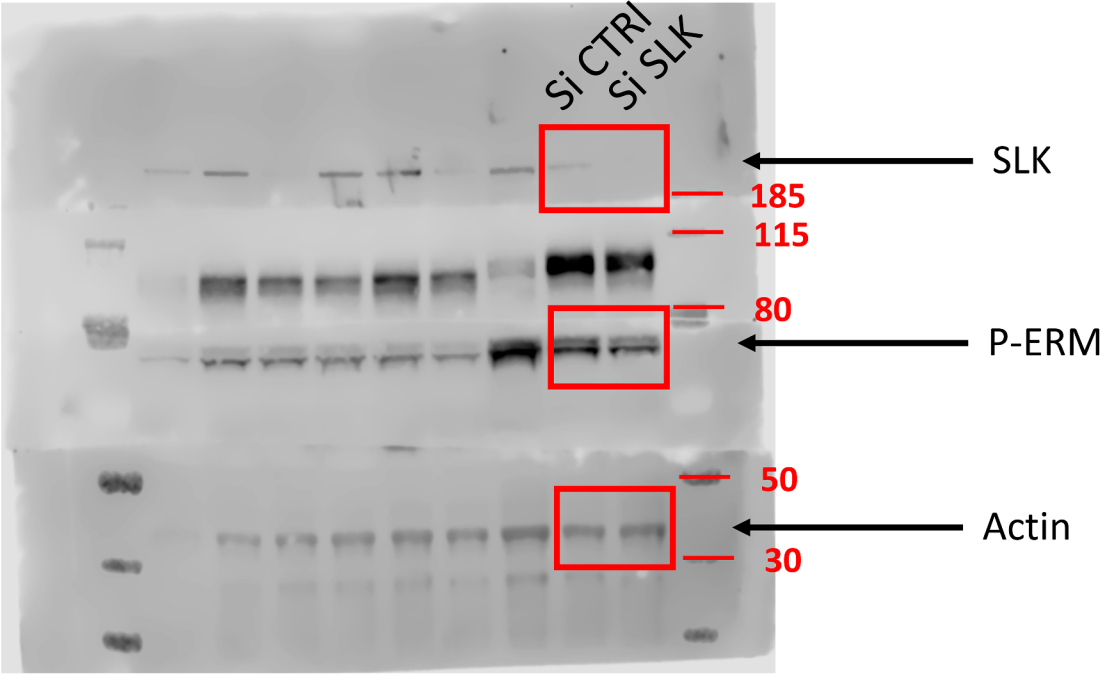

WB\_Figure 6D

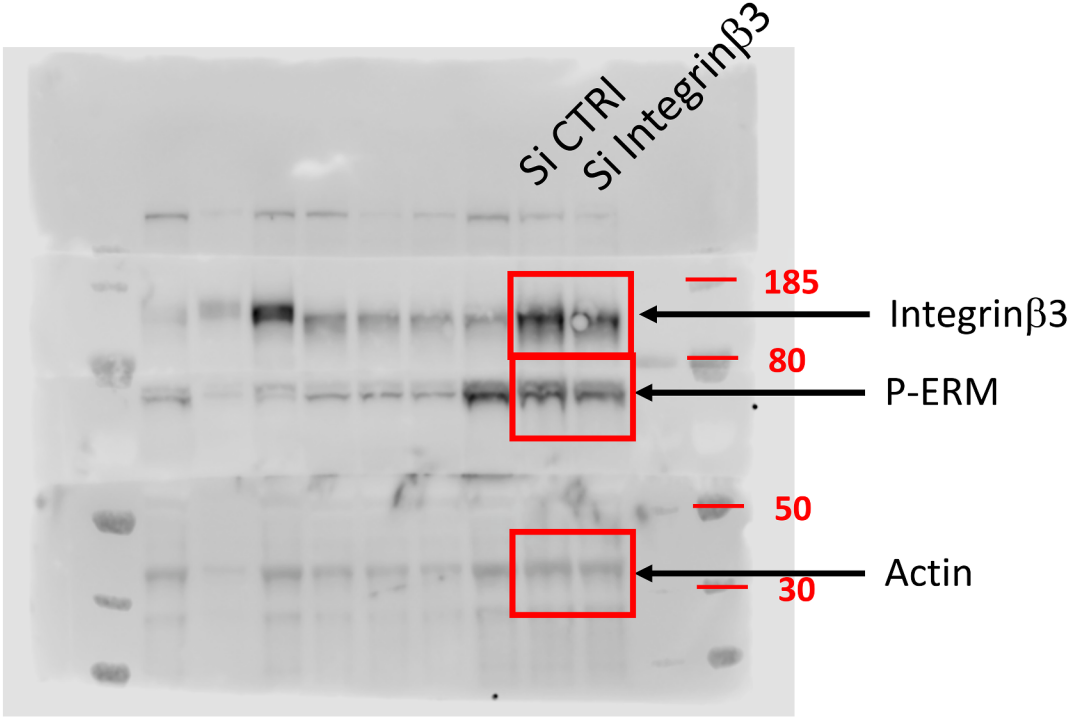

Supplement: SourceData F6 — is the source file for Fig. 6. [file jcb_202409169_sourcedataf6.pdf]

WB\_Supplemental Figure 5D-E

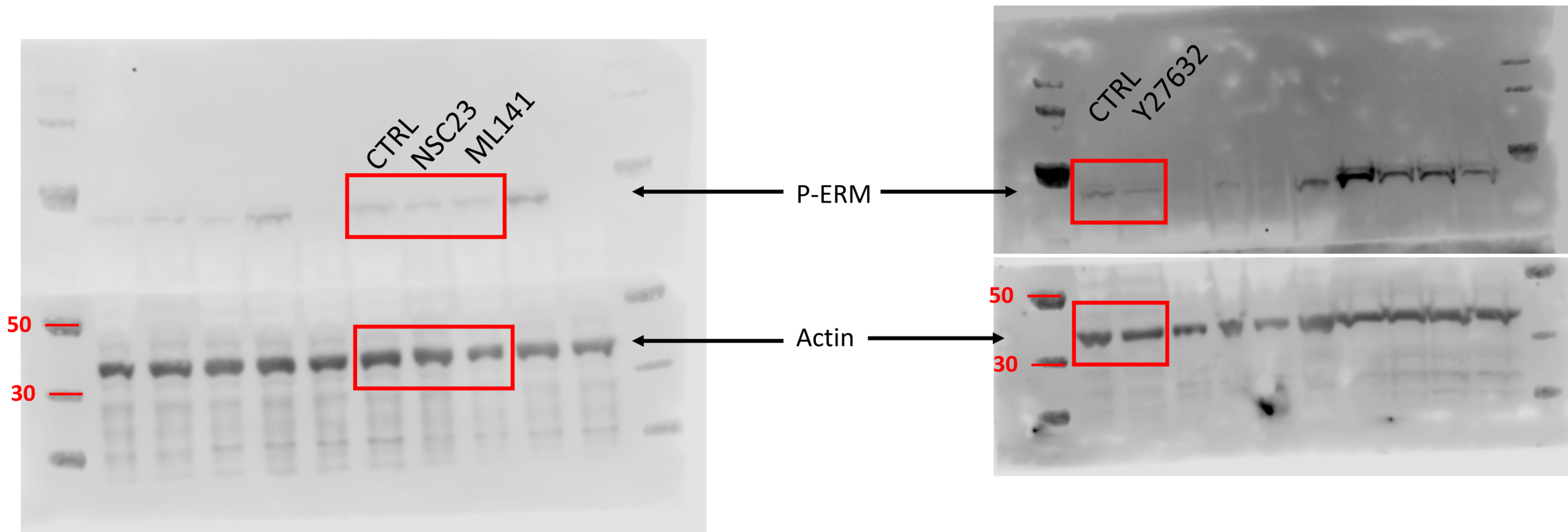

Supplement: SourceData FS5 — is the source file for Fig. S5. [file jcb_202409169_sourcedatafs5.pdf]
