# Supplementary material for: Moesin controls cell–cell fusion and osteoclast function
Source: J Cell Biol. 2025 Oct 27;224(11):e202409169. doi: 10.1083/jcb.202409169 (PMC12558046; doi:10.1083/jcb.202409169)

WB_Supplemental Figure 1A-B

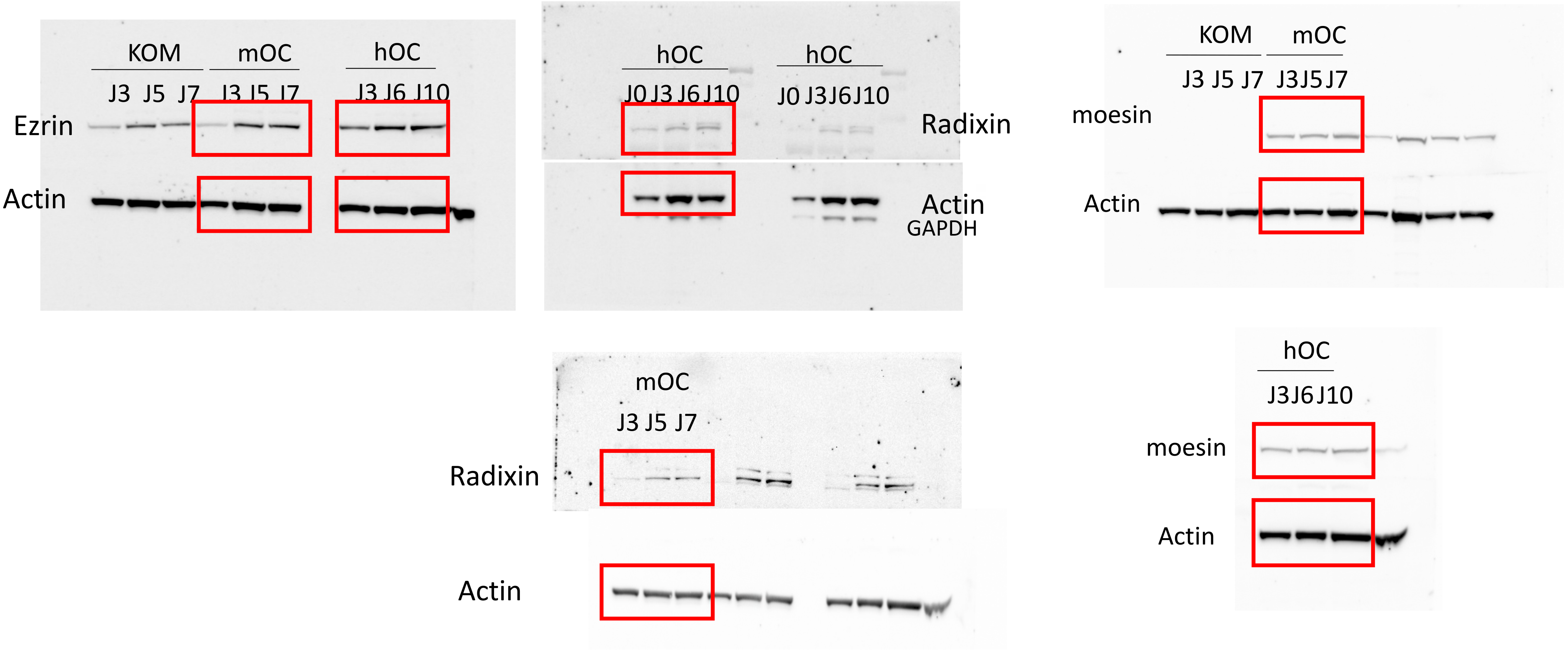

WB_Supplemental Figure 1C

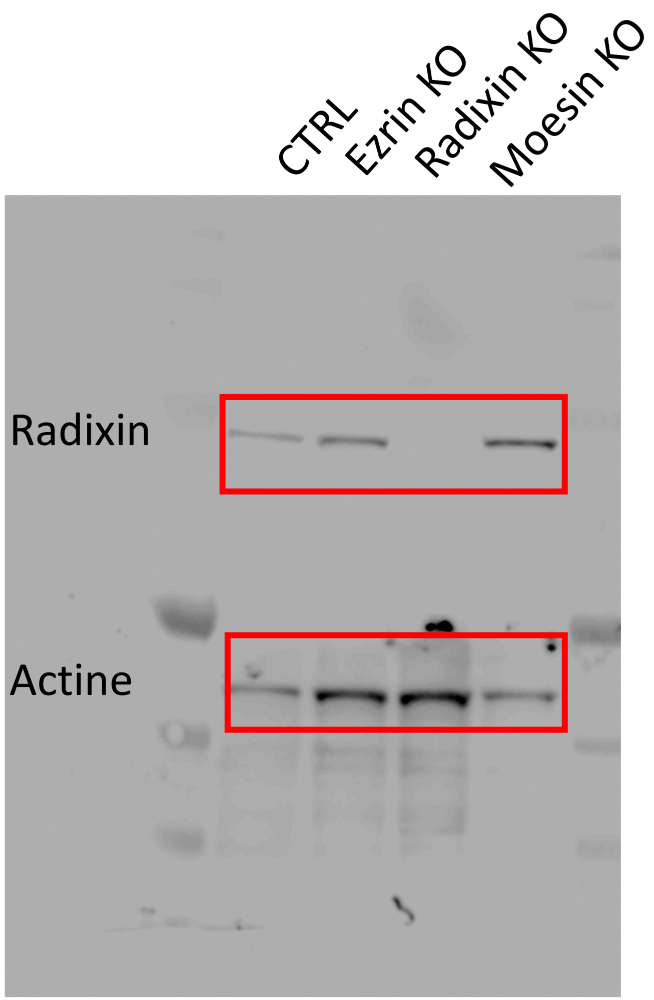

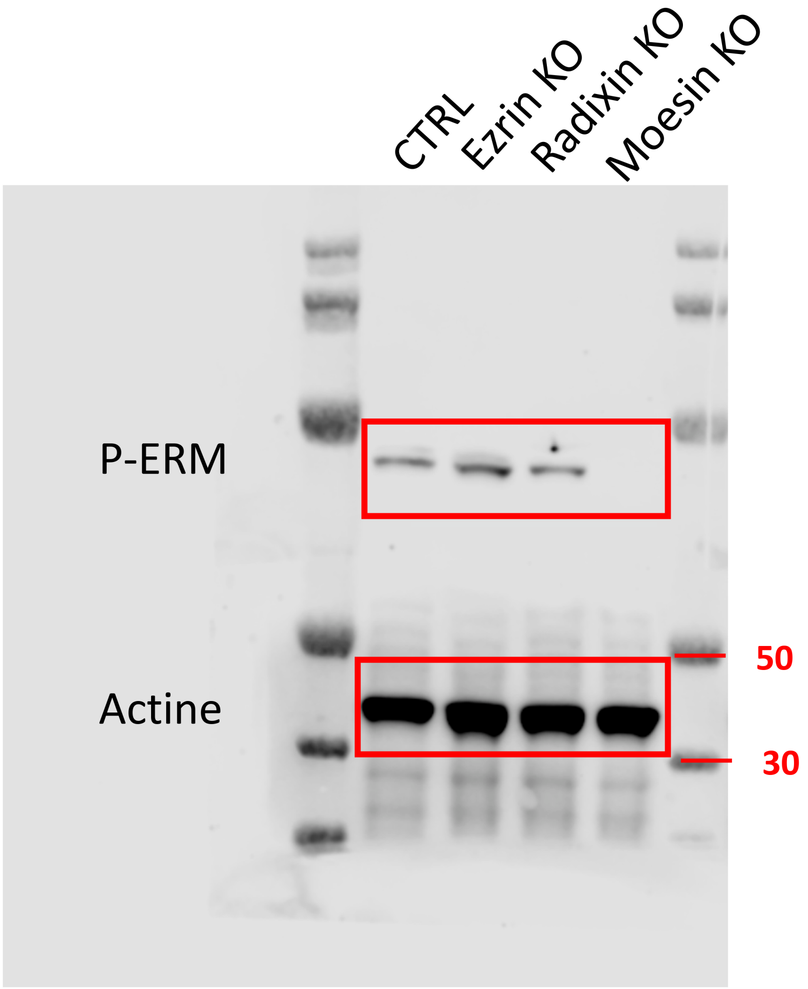

WB_Supplemental Figure 1C

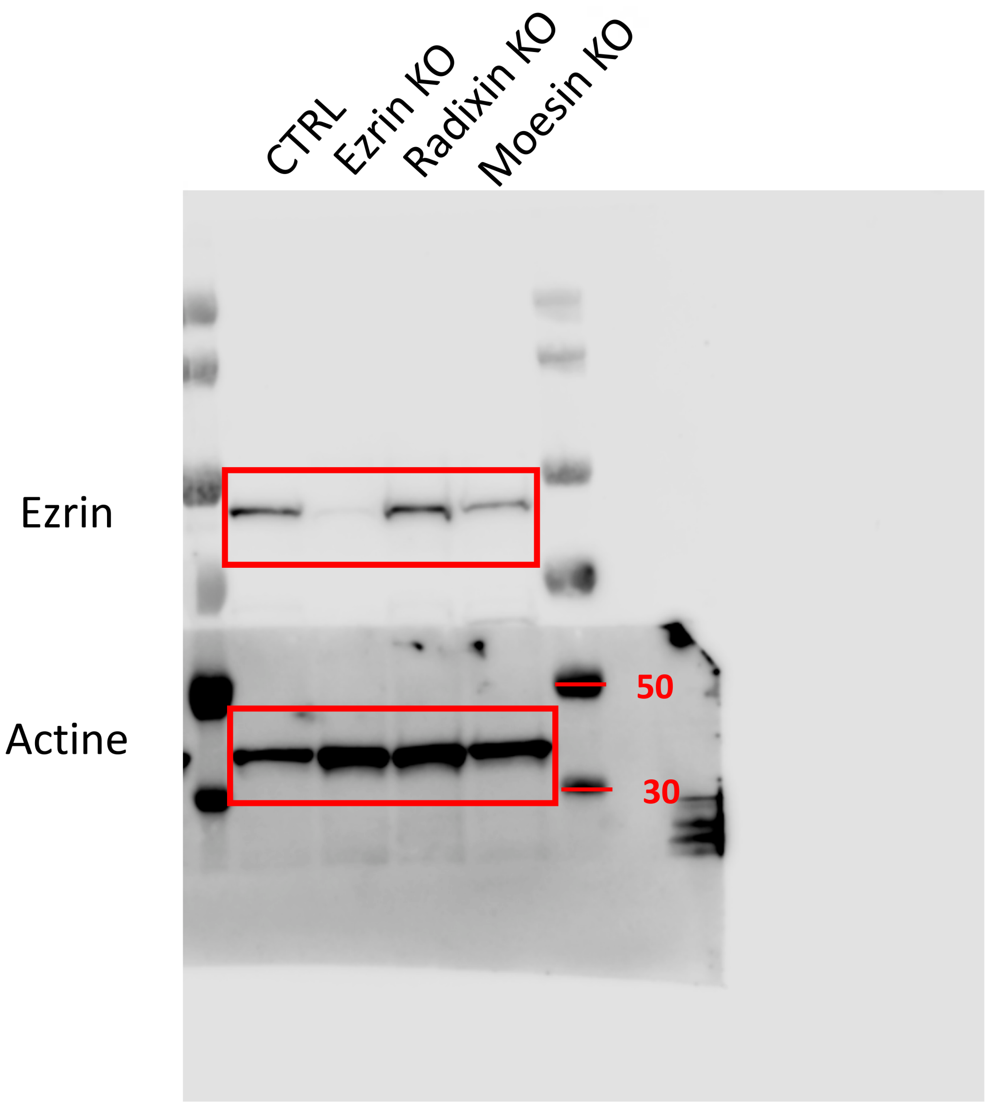

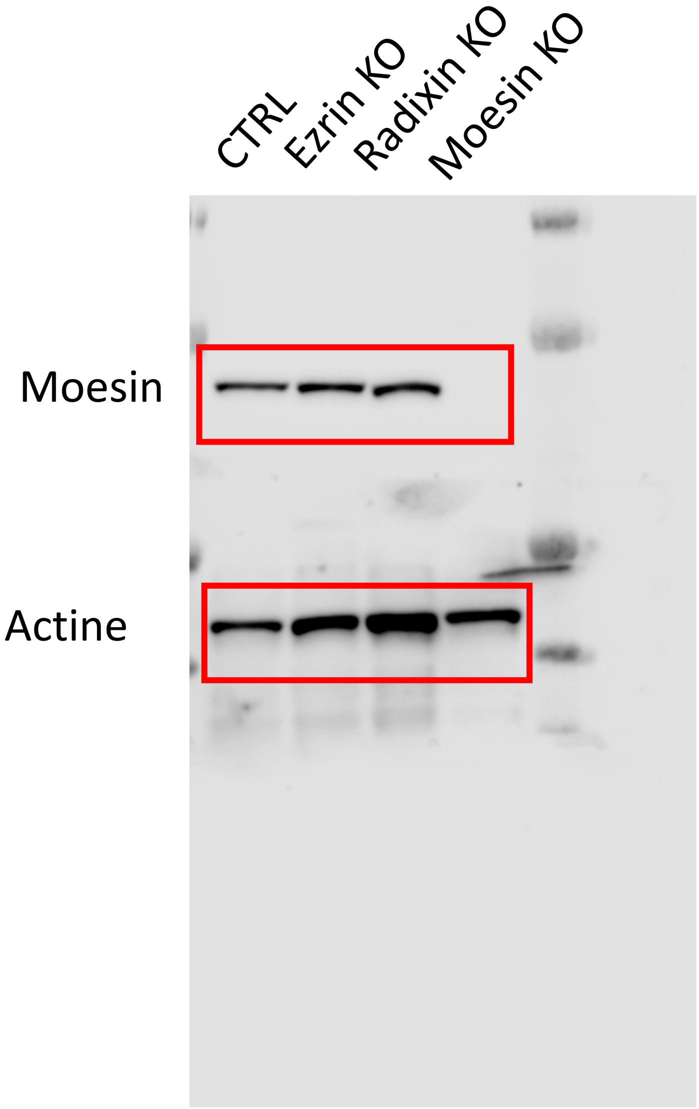

WB_Supplemental Figure 1H

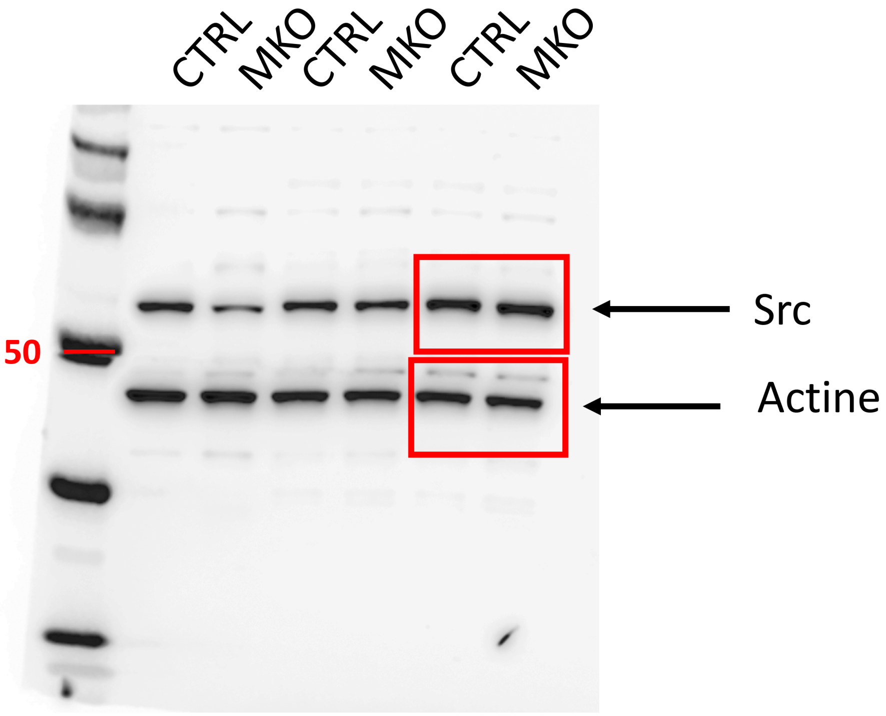

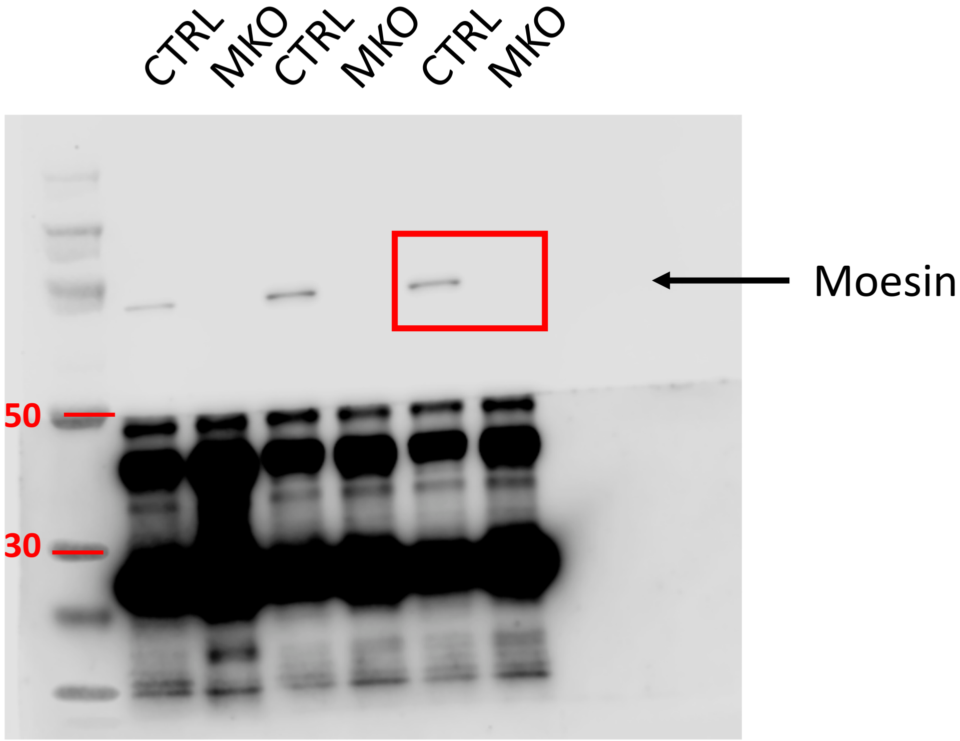

WB_Supplemental Figure 1I

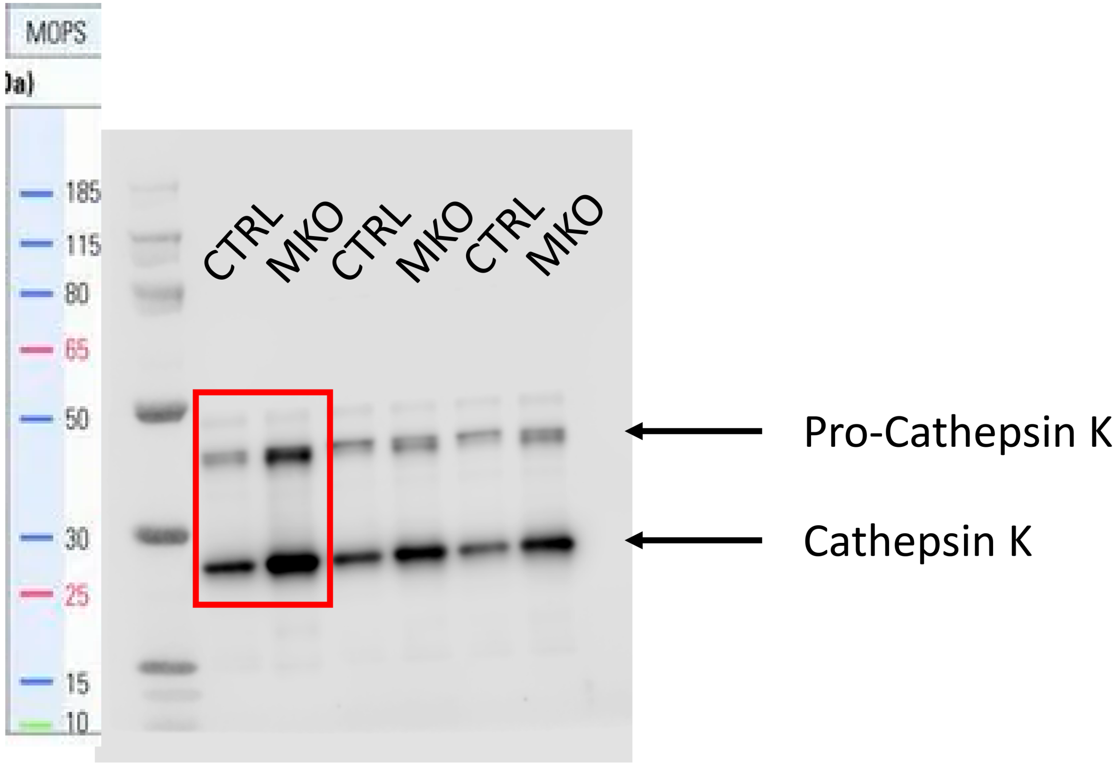

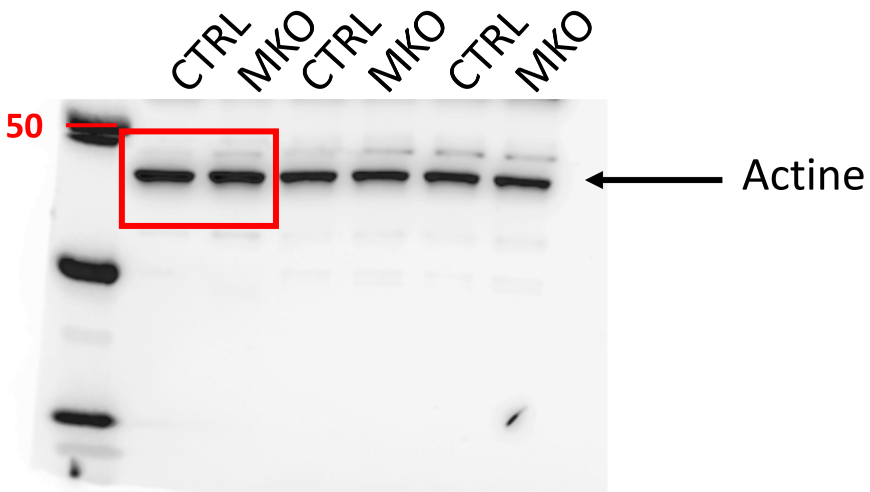

Supplement: SourceData FS1 — is the source file for Fig. S1. [file jcb_202409169_sourcedatafs1.pdf]
