# Supplementary material for: Moesin controls cell–cell fusion and osteoclast function
Source: J Cell Biol. 2025 Oct 27;224(11):e202409169. doi: 10.1083/jcb.202409169 (PMC12558046; doi:10.1083/jcb.202409169)

WB_Supplemental Figure 2A

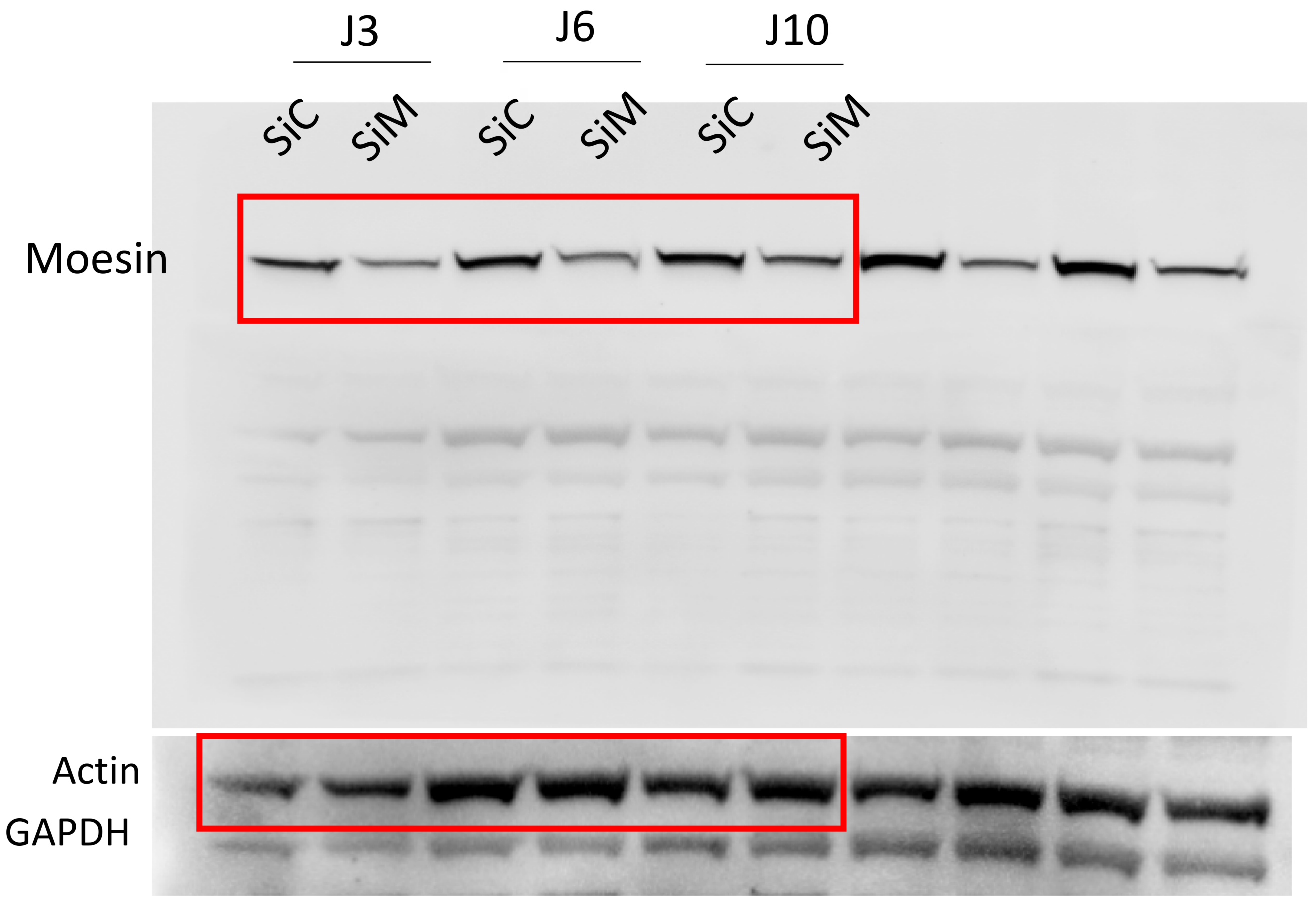

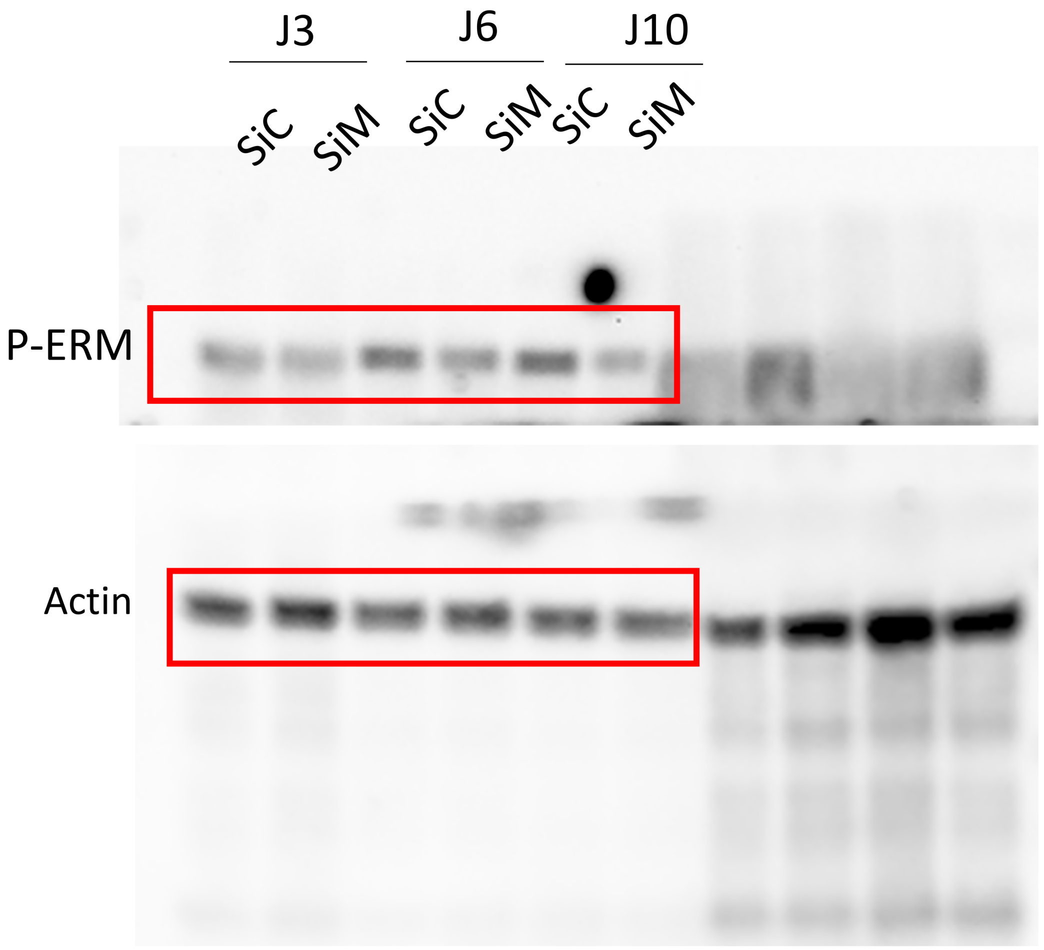

WB_Supplemental Figure 2E

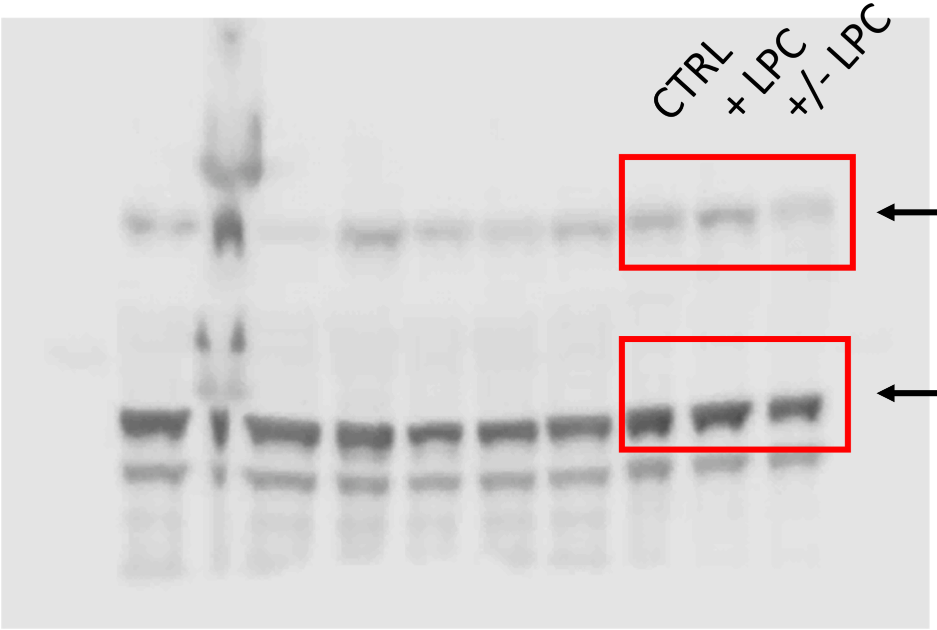

WB_Supplemental Figure 2F

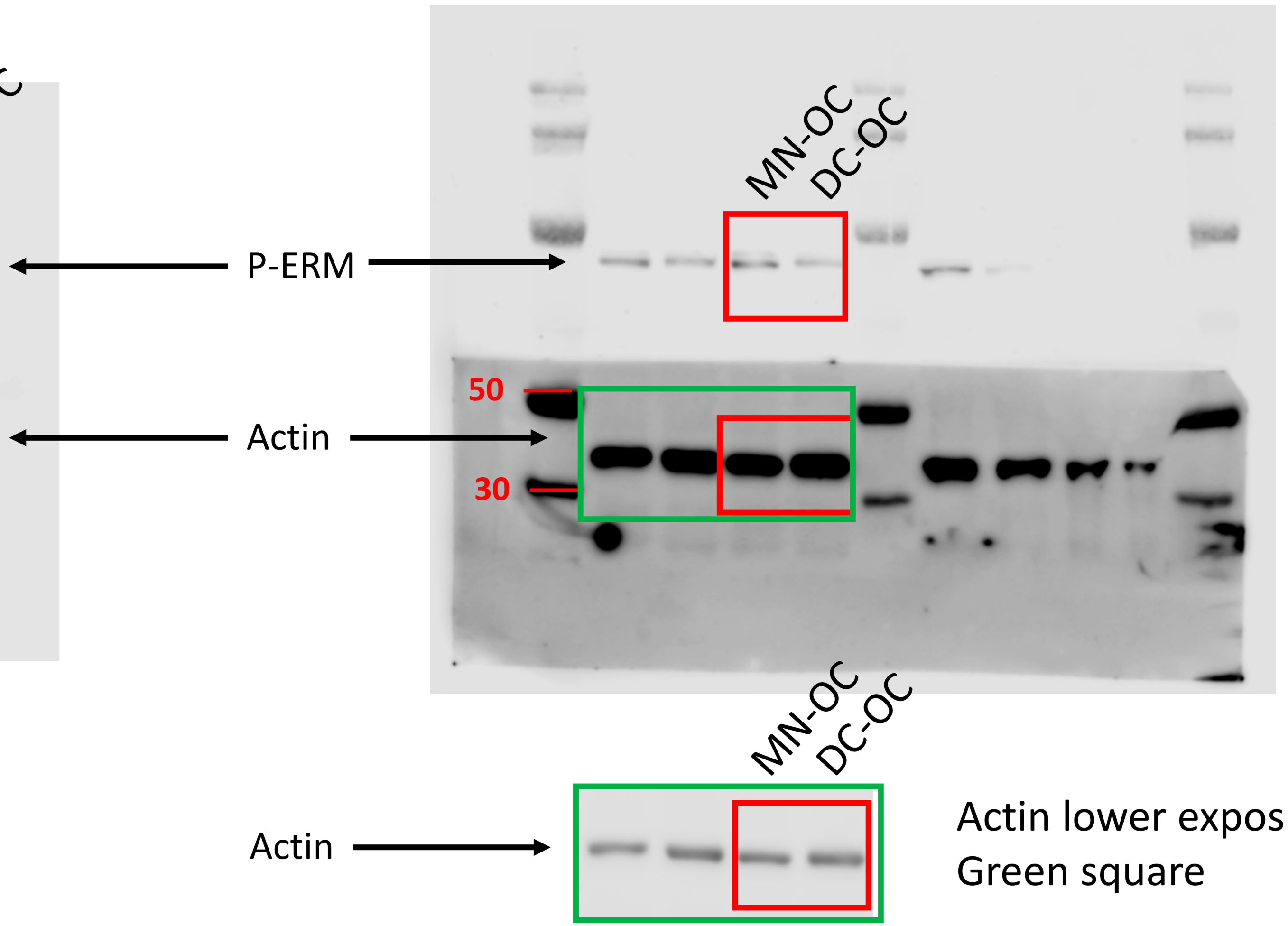

Actin lower exposure (cropped)
Green square

WB_Supplemental Figure 2H

WB_Supplemental Figure 2J

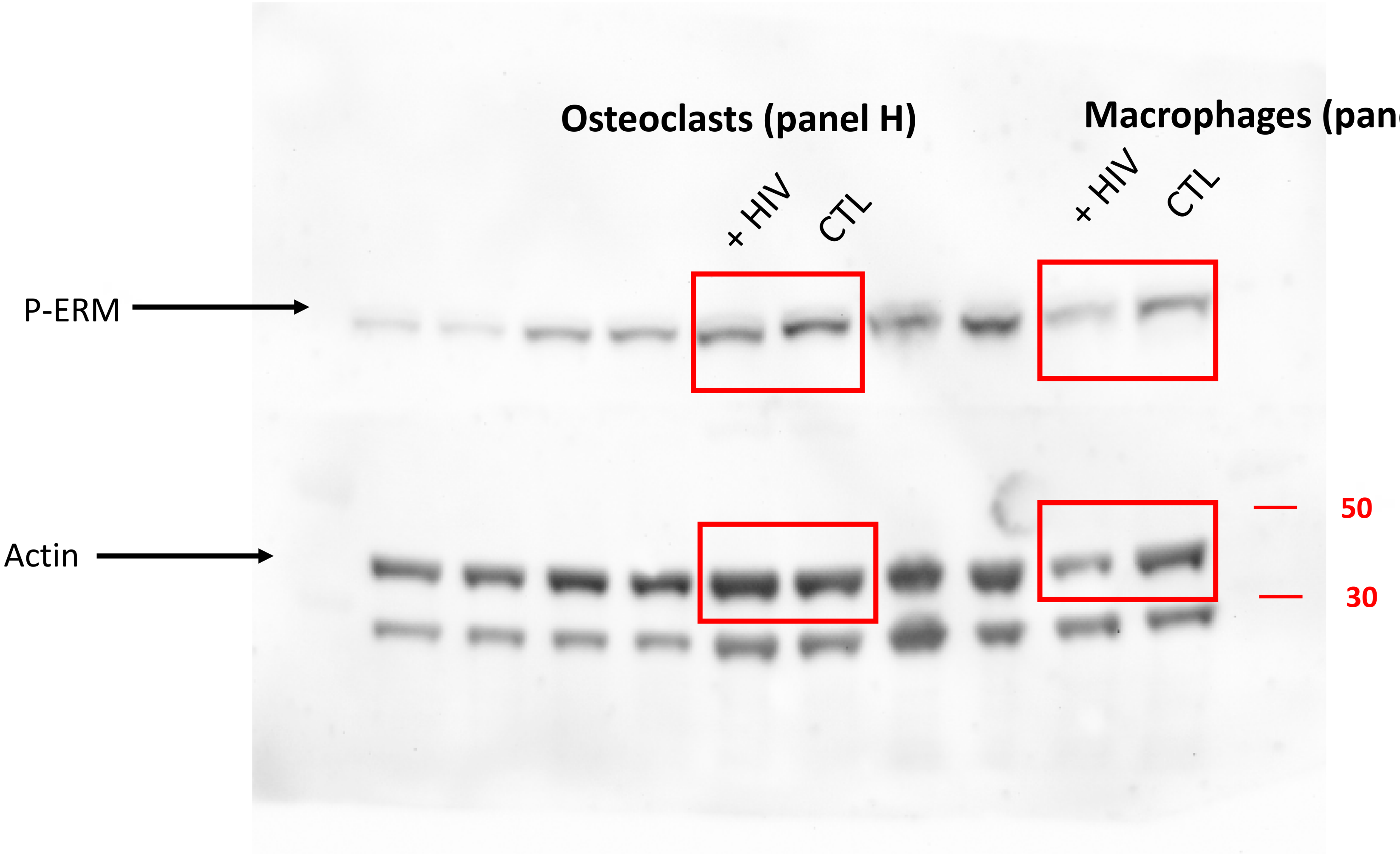

Supplement: SourceData FS2 — is the source file for Fig. S2. [file jcb_202409169_sourcedatafs2.pdf]
